# Manipulation of and Sustained Effects on the Human Brain Induced by Different Modalities of Acupuncture: An fMRI Study

**DOI:** 10.1371/journal.pone.0066815

**Published:** 2013-06-28

**Authors:** Yin Jiang, Hong Wang, Zhenyu Liu, Yuru Dong, Yue Dong, Xiaohui Xiang, Lijun Bai, Jie Tian, Liuzhen Wu, Jisheng Han, Cailian Cui

**Affiliations:** 1 Neuroscience Research Institute, Peking University, Beijing, China; 2 Department of Neurobiology, School of Basic Medical Sciences, Peking University, Beijing, China; 3 Key Laboratory of Neuroscience, The Ministry of Education and Ministry of Public Health, Beijing, China; 4 Department of Magnetic Resonance, General Hospital of Armed Police Forces, Beijing, China; 5 Institute of Automation, Chinese Academy of Sciences, Beijing, China; University of Maryland, College Park, United States of America

## Abstract

The javascript:void(0)manipulation and sustained effects of acupuncture have been investigated in multiple studies, but several findings are inconsistent with one another. One possible explanation for these discrepancies is that different modalities of acupuncture were utilized in these studies. In the present study, we investigated both the manipulation and sustained effects of acupuncture in different modalities, including manual acupuncture (MA), electroacupuncture (EA) and transcutaneous electrical acupoint stimulation (TEAS). MA, EA, TEAS and sensory control stimulation were applied to 18 healthy subjects, and combined block-designed and resting-state fMRI scans were performed. In analyzing these data, the block-designed datasets were used to assess the manipulation effect by employing a modified general linear model. The data from the resting states, before and after stimulation, were used to explore the brain networks involved in the sustained effect. The results showed that the two 1-min stimulation periods produced similar activation patterns in the sensory control with positive activation in the sensorimotor areas and negative activation in the default mode areas. Although similar patterns could be detected in the first stimulation period in MA, EA and TEAS, no positive activation result was observed in the second stimulation period, and EA showed a more extensive deactivation compared to MA and TEAS. Additionally, all three of the modalities of acupuncture stimulation could increase the instinct brain network in rest. A more secure and spatially extended connectivity of the default mode network was observed following MA and EA, and TEAS specifically increased the functional connectivity in the sensorimotor network. The present study suggested that different brain mechanisms might be recruited in different acupuncture modalities. In addition, the findings from our work could provide methodological information for further research into the mechanism of acupuncture.

## Introduction

Acupuncture is a traditional Chinese treatment that has been used in the Orient for thousands of years and is now gaining widespread acceptance as an alternative and complementary treatment in modern medicine [1]. In addition to traditional manual acupuncture (MA), new acupuncture modalities, such as electroacupuncture (EA) and transcutaneous electrical acupoint stimulation (TEAS), are gaining in popularity.

Unlike MA, which uses manual needling at specific acupoints to achieve a therapeutic effect, in EA, electrical pulses are delivered on the needles inserted into the acupoints, and in TEAS, electrical pulses are delivered on the skin of the acupoints via electrode. There is solid evidence that both EA and TEAS have treatment effects on pain [2], [3] and substance abuse [4], [5] in both humans and animal models. In addition, Zhang et al. recently reported that TEAS could increase the success rate for women undergoing embryo transfer [6] and also had the potential to improve autistic behavior in children [7]. Compared to MA, EA is more effective in pain relief [8], [9], and the precision of the simulation parameters ensures high reproducibility for therapeutic effects and research. Additionally, EA without manual manipulation of the needles also saves labor. TEAS has been shown to be as effective as EA in analgesia [10], and with training for nurses and patients, it can be performed even without an acupuncturist. Furthermore, the non-invasiveness of the procedure makes it more acceptable to patients.

Previous studies in animals have shown that acupuncture stimulation could facilitate the release of specific neuropeptides in the central nervous system and elicit profound physiological effects [11]. However, the exploration of acupuncture mechanisms in the human brain was limited by lack of noninvasive methods until the recent development of imaging techniques, particularly functional magnetic resonance imaging (fMRI). Research has mainly focused on two acupuncture effects: the manipulation and sustained effects. A block-designed method has mostly been used for detecting the manipulation effect of acupuncture, and it has generally been accepted that acupuncture deactivates the limbic system and activates sensorimotor areas [12], [13], [14], [15], [16], [17]. Resting-state connectivity has mostly been used to investigate the sustained effect of acupuncture, and increased functional connectivity in the resting brain network following acupuncture has been observed in many studies [18], [19], [20], [21], [22]. However, only one modality of acupuncture was utilized in these works, and varied results were often reported [23]. It is reasonable to wonder whether different modalities of acupuncture could induce different brain activity responses. To the best of our knowledge, only a small number of studies attempted to compare the manipulation effect induced by MA and EA [24], [25], [26], and the brain activation patterns observed in these studies seemed inconsistent. We hypothesize that these discrepancies may be due to small sample sizes and less powerful statistical thresholds.

The aim of the present study is to investigate both the manipulation and sustained effects induced by three popularly utilized acupuncture modalities, namely, MA, EA and TEAS. We used block-designed datasets combined with a modified general linear model (GLM) analysis [27] to observe the manipulation effect. Data from the resting states before and after stimulation were also collected to detect the sustained effect of acupuncture. On the basis of former studies, the default mode network (DMN) and the sensorimotor network (SMN) could be modulated by acupuncture [18], [21], [28]; thus, our exploration of the sustained effect focused on these two networks.

## Materials and Methods

### Subjects

Eighteen healthy, right-handed participants naïve to acupuncture (9 male, mean age-22 years, range-19 to 27) were enrolled in this experiment. Prior to the commencement of the experiment, all subjects signed an informed consent agreement regarding the purpose, procedure and potential risks of this study and were free to withdraw from the experiment at any time. All research procedures were approved by the ethical committee of Peking University.

### Experiment Procedures

At the beginning of the experiments, subjects were told that there were four modalities in the acupuncture treatment and that the purpose of our research was to use fMRI to determine how the modalities changed brain functions. All subjects were recruited to participate in four fMRI scanning sessions, and in each session, the subjects received only one type of stimulation: MA, EA, TEAS or a sensory control. The four sessions were randomized and separated by a minimum of one week.

Acupuncture was performed at acupoint ST-36 on the left leg (Zusanli, located in the tibialis anterior muscle) and was performed by the same experienced and licensed acupuncturist. The needles used in the MA and EA sessions were sterile, disposable, stainless-steel acupuncture needles, which would not distort MR images, measuring 0.22 mm in diameter and 40 mm in length. The needle was inserted in ST-36 with a depth of 1.5–2.5 cm. In the MA session, stimulation was delivered by twisting the needle at 1–2 Hz. In the EA session, in addition to one needle in ST-36, another needle was shallowly inserted (less than 1 cm depth) to a non-acupoint proximal to ST-36. The same locations were attached with electrode slices to the skin surface in the TEAS session. Current was delivered by HANS (Han’s acupoint nerve stimulator, model LH-202H, Neuroscience Research Institute, Peking University, Beijing, China) with a frequency of 2 Hz in both the EA and TEAS sessions. The current intensity for each subject was adjusted to a maximal but comfortable level (2.16±0.20 mA for EA and 23.73±1.71 mA for TEAS). Manual tapping with a 5.88 von Frey monofilament over ST-36 with a 1–2 Hz frequency was utilized in the sensory control session, which is a maneuver that has often been chosen as a control stimulation in acupuncture studies [24], [29]. In all sessions, no sharp pain feeling was allowed.

**Figure 1:**
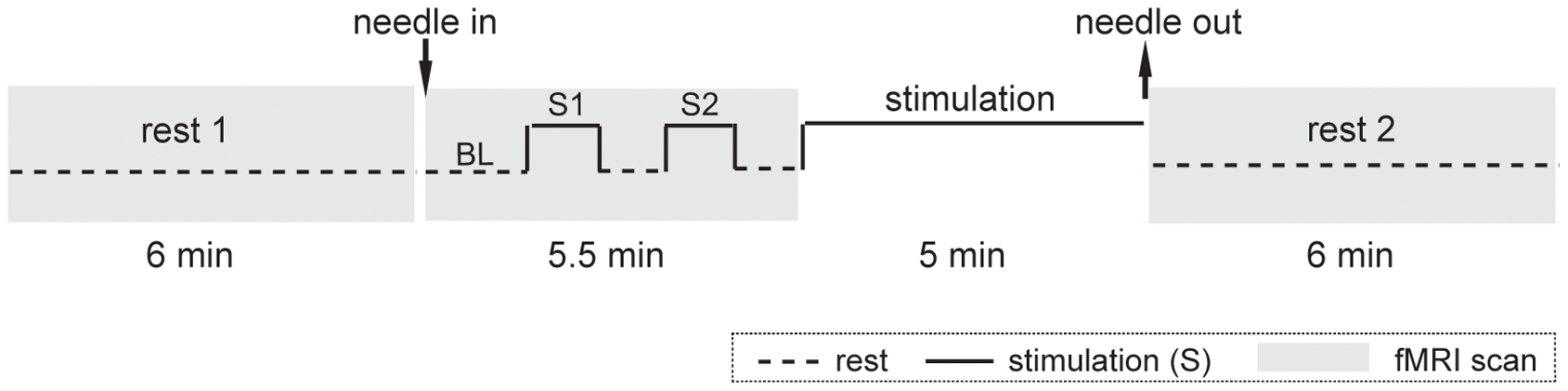
Experimental paradigm. Functional scanning incorporated with three independent runs: two rest runs (rest 1 and rest 2), each lasting 6 min, were separated by a 5.5 min block-designed run and a 5 min stimulation period. The block-designed scanning included two cycles, 1 min stimulation (S1 and S2) and a 1 min rest epoch, preceded by a 1.5 min rest period as baseline (BL). Immediately after the block-designed run, the same modality of stimulation was continued for 5 min without scanning.

Functional scanning was incorporated with three independent runs in each session. Two rest runs, each lasting 6 min, were separated by a 5.5 min block-designed run and a 5 min stimulation period (Fig. 1). During the scanning, subjects lay supine on the scanner bed, wearing ear plugs to suppress scanner noise and with the head immobilized by cushioned supports. They were instructed to keep their eyes closed and their minds clear and to remain awake. In addition, the feelings of *deqi* were collected at the end of the session, including soreness, numbness, fullness, heaviness and dull pain. Subjects were asked to rate each component of the *deqi* feeling they had experienced during the stimulation period using a visual analog scale (VAS) ranging from 0 (none) to 100 (max).

### fMRI Data Acquisition

Functional images were acquired on a Siemens 3T whole-body scanner with a standard whole head coil. Blood oxygenation level-dependent (BOLD) functional imaging was conducted using a T2*-weighted single-shot, gradient-recalled echo planar imaging (EPI) sequence (TE = 30 ms, TR = 2 s, flip angle = 90°, FOV = 250 mm×250 mm). Twenty-nine axial sections, each measuring 4 mm in thickness with 1-mm inter-slices, were collected to encompass the whole cerebrum and cerebellum. Prior to the functional run in the first session, high-resolution structural images of each subject were acquired using a 3D T1-weighted sequence (TR/TE = 2.7s/3.19 ms, FOV = 256 mm×256 mm, flip angle = 7°, slice thickness = 1 mm).

### fMRI Data Analysis

SPM5 software (Wellcome Department of Cognitive Neurology, London, UK) and Group ICA of the fMRI Toolbox (GIFT, http://icatb.sourceforge.net/) were used for the fMRI data analysis. For each run, all the functional images were first realigned to the first one. The image data were further processed with spatial normalization based on the Montreal Neurological Institute (MNI) template and resampled at 2 mm×2 mm×2 mm and spatially smoothed thereafter using a Gaussian Kernel with 6 mm full-width at half maximum (FWHM). Then these data were filtered to reduce the effect of low-frequency drift and high-frequency noise by using a band-pass filter (0.01–0.08 Hz).

To investigate the manipulation effect, GLM was used to analyze the block-designed data. Because the sustained effect of acupuncture has been shown to exist even after a very short period (1 min) of acupuncture stimulation [27], we utilized a modified GLM design matrix that separated different conditions across each subject with regressors coded for the difference between the baseline (BL) and the stimulation period (S1 and S2) (Fig. 1). Further statistical analyses were performed at both the individual level and the group level. In the individual analysis, two *t*-contrasts were defined as S1 minus BL and S2 minus BL. The resulting statistical maps indicated the voxel-wise signal changes for a specific stimulation condition relative to the baseline. These maps from each subject were later used to generate the group map using one sample *t*-test. Statistical significance was thresholded at cluster-level FDR corrected to *P*<0.05, with a cluster size of no less than 15 voxels.

To investigate the sustained effects of acupuncture, independent component analysis (ICA) was used to analyze the rest datasets. Using the Informax ICA algorithm, the smoothed rest data were separated into 40 independent components, and the number was estimated by minimum description length criteria. The DMN or SMN component was identified by spatially sorting the entire components with the corresponding mask [30]. Next, for each subject, the best-fit component was extracted from each individual run. One sample t-test with a significant level of voxel-level FDR corrected to *P*<0.05 was used to examine the group maps for the DMN and SMN, and these maps were made into masks for later comparisons. Paired *t*-tests were performed to determine the differences in the spatial extant of DMN/SMN between rest 1 versus rest 2 for each modality of stimulation, thresholded at voxel-wise of *P*<0.001 uncorrected with 15 continuous voxels within the masks.

## Results

### General Results of Experimental Performance

Sixteen of eighteen consenting volunteers completed the study, and two withdrew. In the functional data processing, data with head movements exceeding 1 mm on any axis or with a head rotation greater than 1° were excluded. In the final cohort, the block-designed datasets included 15 subjects for sensory control, 14 for MA, 15 for EA and 15 for TEAS. Meanwhile, there were 16 subjects for sensory control, 15 for MA, 15 for EA and 14 for TEAS in the rest datasets.

**Figure 2:**
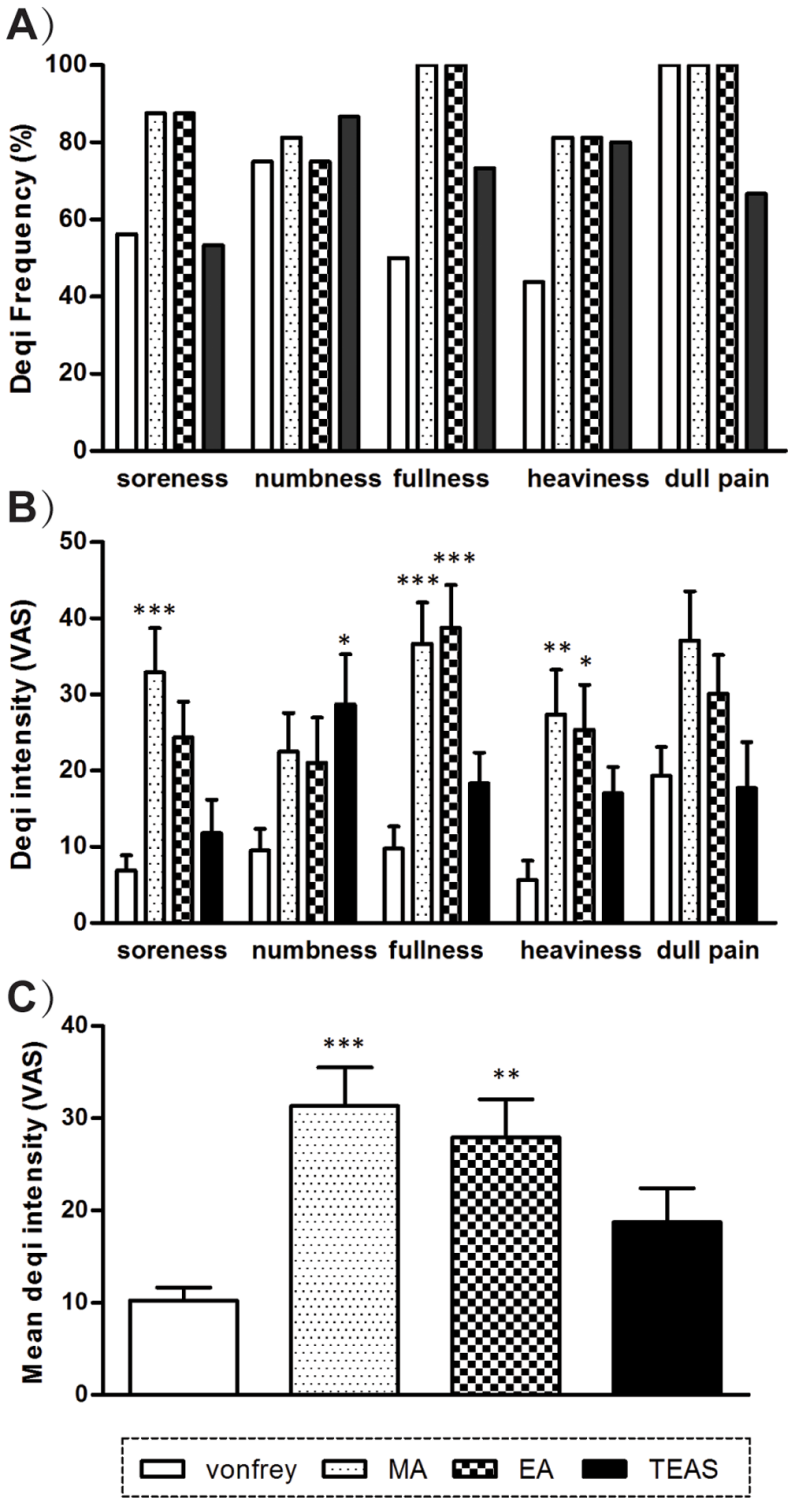
Reports of *deqi* sensations. A) The percentage of subjects who reported having experienced the feelings of *deqi*. B) The intensity of reported sensations measured by an average score (mean±SEM), tested with VAS from 0 to 100. Two-way ANOVA with Bonferroni post-tests were used. C) The mean intensity of all sensations, one-way ANOVA with Tukey’s multiple comparison tests were used. *, *P*<0.05, **, *P*<0.01, ***, *P*<0.001 compared to the sensory control.

The percentage of the subjects who reported *deqi* feelings, including soreness, numbness, fullness, heaviness and dull pain, varied among different types of stimulation (Fig. 2). Compared to sensory control (F_3,295_ = 19.00, *P*<0.001), EA and MA showed higher fullness and heaviness reports. Stronger soreness and numbness feelings were specifically reported in MA and TEAS, respectively, and there were no differences in dull pain (Fig. 2B). The mean intensities of all sensations were also compared, and significant higher mean *deqi* scores were observed in MA and EA, compared to the sensory control (F_3,62_ = 7.252, *P*<0.001) (Fig. 2C).

### Results of the Manipulation Effect

Group results during stimulation on ST-36 included two *t*-contrasts, S1 vs. BL and S2 vs. BL. For sensory control, both S1 and S2 produced signal increases in the sensorimotor area, prefrontal cortex and the cerebellum, and decreased BOLD signals were observed in the precuneus and the precentral gyrus. In addition to these areas, the insula, anterolateral prefrontal cortex, striatum and the middle temporal gyrus showed positive activation during S1 (Table 1). During MA on ST-36, S1 also increased the BOLD responses in the sensorimotor area, the anterolateral prefrontal cortex and the middle temporal gyrus. However, there was neither positive nor negative activation during S2 (Table 2). EA only activated the insula and the cerebellum in S1 but produced extensive signal decreases in the sensorimotor area, the limbic system, and other cortical regions such as the prefrontal cortex, superior temporal gyrus and the precuneus during S2 (Table 3). For TEAS, in addition to similar activations as in the sensory control, the premotor cortex, the thalamus and the parahippocampal gyrus showed specific signal changes in S1. There was no positive activation above the statistical threshold in S2, and deactivations were observed in the premotor area and the precuneus (Table 4).

**Table 1:** Regions of activation for group analysis of sensory control in different stimulation periods.

		stimulation 1 vs. baseline					stimulation 2 vs. baseline				
region	side	t value	coordinate (MNI)			voxels	t value	coordinate (MNI)			voxels
			x	y	z			x	y	z	
**positive activation (stimulation>baseline)**											
primary and secondary somatosensory area	L	7.09	−56	−24	24	835	6.83	−56	18	30	1091
	R	7.19	60	−28	30	1009	7.28	52	−42	58	1001
insula	R	6.62	32	−2	16	145					
lateral prefrontal cortex	L	4.90	−48	12	16	71	6.51	−42	8	26	532
	R	6.49	54	10	32	624	8.26	46	16	2	231
anterolateral prefrontal cortex	L	4.47	−38	28	6	72					
	R	5.46	40	44	0	155					
middle temporal gyrus	R	5.23	54	−62	2	129					
striatum	R	8.20	20	−14	−8	48					
cerebellum	L	8.06	−22	−66	−44	860	7.48	−22	−72	−50	474
	R	5.06	14	−74	−46	71					
**negative activation (stimulation<baseline)**											
precuneus	L	−5.77	−16	−96	25	453	−7.22	10	−60	22	360
	R	−5.34	20	−95	25	299					
precentral gyrus	L	−5.64	−26	−30	64	220	−6.89	−28	−16	72	98
	R	−4.50	42	−26	56	77					

L, left; R, right.

**Table 2:** Regions of activation for group analysis of MA in different stimulation periods.

		stimulation 1 vs. baseline					
region	side	t value	coordinate (MNI)			voxels	
			x	y	z		
**positive activation (stimulation>baseline)**							
primary and secondary somatosensory area	L	6.23	−48	−26	38	443	
	R	8.48	58	−22	26	665	
anterolateral prefrontal cortex	R	6.38	42	56	10	95	
middle temporal gyrus	R	6.81	52	−52	−4	176	
**negative deactivation (stimulation<baseline)**							
no regions above threshold							

L, left; R, right. There was no statistically significant region in comparison of ‘stimulation 2 vs. baseline’.

**Table 3:** Regions of activation for group analysis of EA in different stimulation periods.

		stimulation 1 vs. baseline					stimulation 2 vs. baseline				
region	side	t value	coordinate (MNI)			voxels	t value	coordinate (MNI)			voxels
			x	y	z			x	y	z	
**positive activation (stimulation>baseline)**											
insula	R	4.68	48	8	0	129					
cerebellum	L	6.52	−16	−68	−44	143					
**negative activation (stimulation<baseline)**											
postcentral gyrus	L						−6.62	−12	-46	64	460
parahippocampal gyrus	R						−6.33	14	−12	−24	186
superior temporal gyrus	R						−5.38	54	−58	16	135
supplementary motor area	L						−4.64	−4	−18	66	90
premotor cortex	R						−6.22	22	2	68	87
precuneus	R						−6.20	4	−54	46	209
medial prefrontal cortex	R						−5.94	6	52	50	101
dorsal anterior cingulate cortex	L,R						−4.89	6	10	34	193

L, left; R, right.

**Table 4:** Regions of activation for group analysis of TEAS in different stimulation periods.

		stimulation 1 vs. baseline					stimulation 2 vs. baseline				
region	side	t value	coordinate (MNI)			voxels	t value	coordinate (MNI)			voxels
			x	y	z			x	y	z	
**positive activation (stimulation>baseline)**											
primary and secondary somatosensory area	L	6.64	−48	−40	26	544					
	R	7.87	68	−22	28	591					
postcentral gyrus	R	5.26	12	−48	74	107					
supplementary motor area	R	6.01	8	−20	68	92					
anterolateral prefrontal cortex	L	5.00	−38	46	−12	112					
	R	5.52	42	42	−10	145					
middle temporal gyrus	R	5.07	−52	−58	4	62					
thalamus	R	5.73	16	−14	−4	727					
striatum	L	4.69	−24	2	20	118					
cerebellum	L	6.36	−30	−62	−48	88					
	R	5.27	26	−66	−26	88					
**negative activation (stimulation<baseline)**											
precentral gyrus	R	−5.82	38	−16	64	202					
premotor cortex	L						−5.87	−22	12	70	188
	R						−6.68	32	10	64	257
parahippocampal gyrus	L	−5.37	−20	−8	−30	87					
	R	−5.45	30	−20	−26	200					
precuneus	L						−4.71	−8	−64	58	285
	R						−5.25	4	−46	48	90

L, left; R, right.

### Results of the Sustained Effect

**Figure 3:**
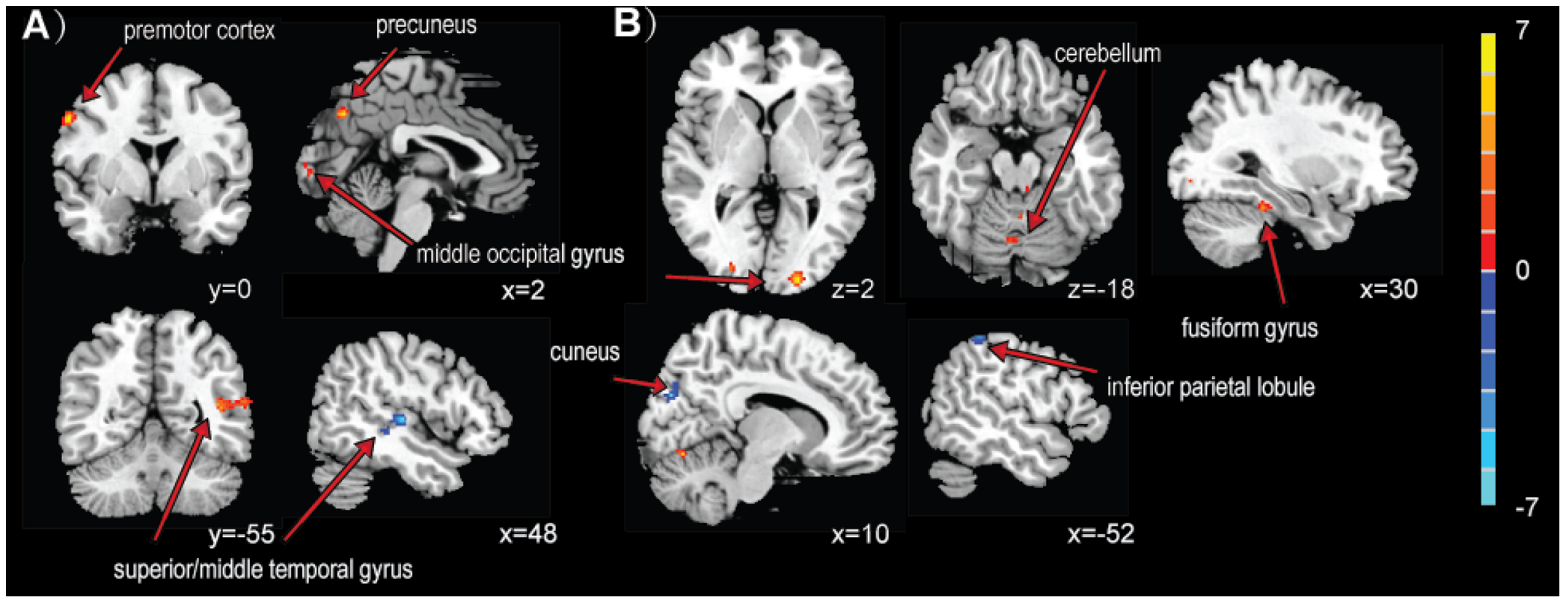
Changes in functional connectivity of the DMN following MA (A) or EA (B). Using paired *t*-test. The threshold of display was set to voxel-wise *P*<0.001 uncorrected with at least 15 contiguous voxels. The colored bar indicates T-values. The group maps for the DMN before and after MA/EA stimulation were shown in Fig.S1A.

The group maps of the DMN in the resting state consistently demonstrated spatial distribution with the DMN mask (Fig.S1A), including in the posterior cingulate, precuneus, medial prefrontal cortex and the inferior parietal lobule. Increased connectivity of this network was observed in the precuneus, middle occipital gyrus, temporal gyrus and the premotor cortex following MA stimulation. Additionally, the middle occipital gyrus, fusiform gyrus and the cerebellum also showed increased connectivity in EA. Decreased connectivity was found in the superior temporal gyrus following MA, in the inferior parietal lobule following EA and in the cuneus after EA and TEAS. There was no connectivity change in the sensory control (Table 5 and Fig. 3).

**Table 5:** Brain regions in the DMN modulated by different modalities of acupuncture.

		MA					EA					TEAS				
Region	side	t value	coordinate (MNI)			voxels	t value	coordinate (MNI)			voxels	t value	coordinate (MNI)			voxels
			x	y	z			x	y	z			x	y	z	
**Rest 1<Rest 2**																
premotor cortex	R	5.91	50	0	40	22										
middle temporal gyrus	L	4.82	−42	−54	12	19										
superior temporal gyrus	L	5.49	−62	−56	14	15										
middle occipital gyrus	L	5.89	−24	−86	−8	34	5.84	−20	−96	2	71					
fusiform gyrus	R						4.87	30	−38	−24	23					
precuneus	R	6.08	2	−70	38	50										
cerebellum	L						5.40	−8	−40	−24	16					
	R						5.29	2	−58	−18	29					
**Rest 1>Rest 2**																
superior temporal gyrus	R	−6.28	48	−20	4	28										
inferior parietal lobule	L						−4.87	−52	−42	56	21					
Cuneus	L											−4.50	−12	−104	4	28
	R						−6.39	10	−80	26	48					

L, left; R, right. There was no statistically significant region in comparison of ‘sensory control’.

**Figure 4:**
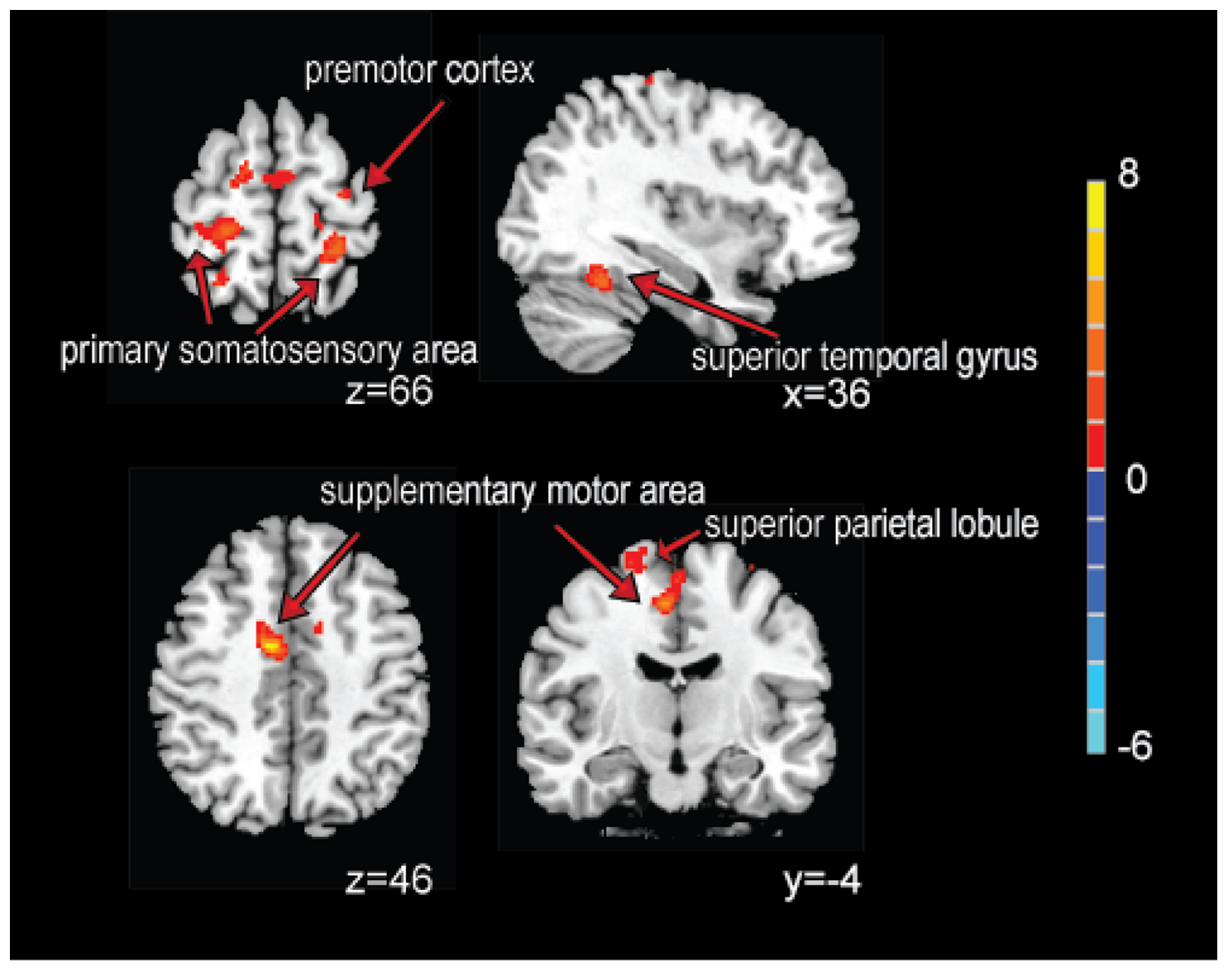
Changes in functional connectivity of the SMN following TEAS. Using paired *t*-test. The threshold of display was set to voxel-wise *P*<0.001 uncorrected, with at least 15 contiguous voxels. The colored bar indicates T-values. The group maps for the SMN before and after TEAS were shown in Fig.S1B.

The group maps of the SMN during rest were also consistently spatially distributed with a predefined mask (Fig.S1B) and included the pre- and post-central gyrus, supplemental motor area and the secondary somatosensory area. MA, EA and the sensory control showed decreased connectivity in this network. Decreases were observed in the primary somatosensory area and the premotor cortex after MA, in the cuneus after EA, in the premotor cortex and the supplementary motor area after sensory control. However, TEAS predominantly increased connectivity in several regions, including the primary somatosensory area, the premotor cortex, the dorsal anterior cingulate cortex, the supplementary motor area, the superior temporal and the parietal lobule (Table 6 and Fig. 4).

**Table 6:** Brain regions in the SMN modulated by different modalities of acupuncture.

		sensory control					MA					EA					TEAS				
Region	side	t value	coordinate (MNI)			voxels	t value	coordinate (MNI)			voxels	t value	coordinate (MNI)			voxels	t value	coordinate (MNI)			voxels
			x	y	z			x	y	z			x	y	z			x	y	z	
**Rest 1<Rest 2**																					
primary somatosensory area	L																6.09	−36	−34	60	102
	R																5.17	22	−34	66	102
premotor cortex	L																4.91	−30	−16	68	19
dorsal anterior cingulate cortex/supplementary motor area	R																7.88	10	−4	46	243
superior temporal gyrus	R																5.57	36	−52	−22	28
superior parietal lobule	R																4.14	22	−12	70	11
**Rest 1>Rest 2**																					
primary somatosensory area	R						−5.49	42	−24	64	53										
premotor cortex	L						−6.27	−48	−12	46	21										
	R	−5.15	64	−6	34	20															
supplementary motor area	R	−4.64	4	8	62	18															
Cuneus	L											−5.25	−16	−80	16	19					

L, left; R, right.

## Discussion

Although the manipulation effect of acupuncture on human brain activity has been studied for a long time, most of these studies have used block-designed datasets with typical GLM contrast analysis [15], [17], [24], [31]. However, it is well-known that the sustained effects of acupuncture could last for a long period of time after removing the needles [27], [32], [33]. Thus, the typical GLM analysis with block-design is inappropriate for identifying the manipulation effect on human brain activity from acupuncture. In the present study, only the 1.5 min rest period before stimulation was defined as the baseline (Fig. 1), and a modified GLM design matrix was used [27]. Thus, the manipulation effect can be observed without baseline contamination from other rest periods after acupuncture.

In our study, the sensory control stimulation induced similar brain activation during S1 and S2. The BOLD signal increases were mainly distributed in the sensory-motor areas and the lateral prefrontal cortex. The negative activation was observed in DMN (Table 1). These findings were mostly reported by other studies using 5.88 von Frey monofilaments as sensory controls [14], [24], [33]. In addition, the activation during S1 was more widespread than during S2, which could be attributed to habituation, a progressive decrease in the physiological response to a repeating stimulus that is neither rewarding nor harmful [34]. In contrast, for MA, EA and TEAS, group results during S1 and S2 displayed distinct patterns of activation. Similar positive activation in the sensory-motor area was observed during S1, but the BOLD responses during S2 trended to a negative activation pattern. In Table 2–4, no positive activation could be found in each of the three types of acupuncture stimulation during S2, and EA produced predominantly negative BOLD responses in brain regions, including the sensory-motor areas, the limbic system and other cortical gyri. It is in keeping with the findings that acupuncture stimulation evokes deactivation in the limbic-paralimbic-neocortical network [12], [13]. Although MA and TEAS exhibited absent or reduced deactivation during this period, we did observe sub-thresholded decreases in BOLD signal changes. Studies using a less strict level of significance also observed a trend of deactivation instead of activation during the S2 period in the MA group [27]. For pain-relief, EA has been shown to be more effective than MA, and TEAS was equally effective as EA [8], [9]. Furthermore, Napadow et al. reported that EA induced more widespread fMRI signal changes than MA when a traditional GLM with block design was used [24]. The current findings indicate that, inconsistent with the sensory habituation in the control, the manipulation effect of all the three modalities of acupuncture stimulation was an early somatosensory activation with later cortical-subcortical deactivation, and EA produced more obvious deactivation than MA and TEAS.

Recently, more studies have paid close attention to the sustained after-effects of acupuncture by comparing the resting state connectivity before and after acupuncture. To the best of our knowledge, most of these studies used short periods of stimulation of less than 6 min [20], [21], [22], [35], which does not fully model the clinical effect produced by relatively longer periods of acupuncture [3]. An early study from our research group revealed a time-curve for the analgesic effect of MA in healthy human beings, and the skin pain threshold started to increase after approximately 10 min of treatment [36]. For this reason, 5 more minutes of stimulation were added after the block to make our model more appropriate for the explanation of the mechanism of potential acupuncture treatment effects.

Interestingly, in the present study, the resting state network following TEAS stimulation displayed a different pattern of connectivity changes than MA and EA. As shown in Table 5 and Fig. 3, the modulating effect of MA and EA is predominantly through the DMN, whereas a more secure and spatially extended connectivity of the SMN was specifically detected in the post-TEAS rest (Table 6 and Fig. 4). Similar increased connectivity between the DMN with other brain regions including the temporal, occipital and frontal cortex were also reported in other studies using MA [21], [28], [37] and EA [38]. This modulatory effect is speculated to be potent in treating diseases with dysfunctional DMN, such as pain, substance abuse and Alzheimer’s disease [39], [40], [41], [42], [43]. Additionally, a recent study reported that although EA had a better analgesic effect than MA, a sustained effect was better produced by MA [9], which might explain the more extended increased connectivity in cortical regions induced by MA in our work. To the best of our knowledge, there has been little research investigating the sustained effect following TEAS, and in this study, we first discovered this specific SMN modulating effect. Dhond et al. reported that MA could also increase the functional connectivity in SMN in several regions by acupuncture in PC-6, but the predominant changes were still focused on the DMN [21]. Recently, long-term transcutaneous electrical nerve stimulation (TENS) was shown to be effective in reorganizing the motor cortex in a neurologically intact human, which highlights the potential benefit of sensory training by TENS as a useful complementary therapy in neurorehabilitation [44]. Thus, we surmised that TEAS might be specifically sensitive in the SMN, and this transcutaneous acupuncture might be more suitable for treating diseases with sensory dysfunction. It is notable that our recent work showed that when the stimulation period lasts 30 min, in addition to the SMN modulatory effect, TEAS could also increase the functional connectivity in the DMN [18]. This finding indicated that the effect of acupuncture may have been dependent on the duration of the stimulation and that the treatment time should be considered to be an important factor for studies on the mechanisms of acupuncture.

The sensations of *deqi* were different in the three modalities of acupuncture stimulation; MA and EA produced stronger *deqi* sensations of fullness and heaviness than did the control (Fig. 2), and stronger soreness was also reported in MA. Interestingly, unlike MA and EA, TEAS specifically induced more reports of numbness. Because the *deqi* sensation is considered to be related to the clinical efficacy in traditional Chinese medicine [45], [46], our results suggested that different types of acupuncture treatment, especially the transcutaneous and invasive acupuncture, might have varied treatment effects, and further studies are required to support this speculation. Moreover, no difference was observed in dull pain sensation between the acupuncture and control groups, which confirmed *deqi* as a multiple-feeling sensation more than only pain [24], [47].

Several limitations in this study should be noted. Although we observed differences among different acupuncture modalities in *deqi* sensation and in the effects on brain activities by using fMRI, less information about autonomic response was collected (for instance, heart rate and skin conductance). Napadow et al. recently reported that different brain responses underling MA stimulation may be related to differential autonomic outflows and may result from heterogeneity in evoked sensations [29]. In addition, a recent report by Florian et al. provided an insight into the linkage of the *deqi* sensation, autonomic responses and the potential of therapeutic effect [48]. Thus, it is possible that the differences in brain activity changes induced by different acupuncture modalities might also have a relationship with the autonomic responses. To study this possibility further, combined fMRI and autonomic response measurements are needed. It also should be noted that the present study focused primarily on changes in brain activity induced by acupuncture in healthy subjects and could only provide clues in exploring the mechanisms of acupuncture treatment. Further studies in patients would provide more convincing evidence of the differences among these modalities of acupuncture. Also, the significant threshold of the paired-*t* test was without FDR corrected, further researches with large sample size may conquer this limitation.

In summary, the current study suggests that although different modalities of acupuncture could be clinically effective, the underlining mechanisms might be varied, and acupuncture in differential modalities might have treatment potentials for specific dysfunctions. In addition, the findings of our research could supply methodological information for further studies examining the mechanism of acupuncture.

## Supporting Information

Figure S1 **Group maps for the DMN and the SMN, before and after acupuncture stimulation.** The best-fit components were selected by using the templates of the DMN and SMN shown in the right line of the graph. The group results of (A) the DMN and (B) the SMN components decomposed by ICA included the pre-MA rest/post-MA rest, the pre-EA rest/post-EA rest and the pre-TEAS rest/post-TEAS rest. The threshold of one sample *t*-test was set as FDR corrected, *P*<0.05, with at least 10 continuous voxels in all group statistics. Color bar indicates T-values.
(DOC)
