# Supplementary material for: Manipulation of and Sustained Effects on the Human Brain Induced by Different Modalities of Acupuncture: An fMRI Study
Source: PLoS One. 2013 Jun 28;8(6):e66815. doi: 10.1371/journal.pone.0066815 (PMC3696086; doi:10.1371/journal.pone.0066815)
Supplement: Figure S1 — Group maps for the DMN and the SMN, before and after acupuncture stimulation. The best-fit components were selected by using the templates of the DMN and SMN shown in the right line of the graph. The group results of (A) the DMN and (B) the SMN components decomposed by ICA included the pre-MA rest/post-MA rest, the pre-EA rest/post-EA rest and the pre-TEAS rest/post-TEAS rest. The threshold of one sample t-test was set as FDR corrected, P<0.05, with at least 10 continuous voxels in all group statistics. Color bar indicates T-values. (DOC) [file pone.0066815.s001.doc]

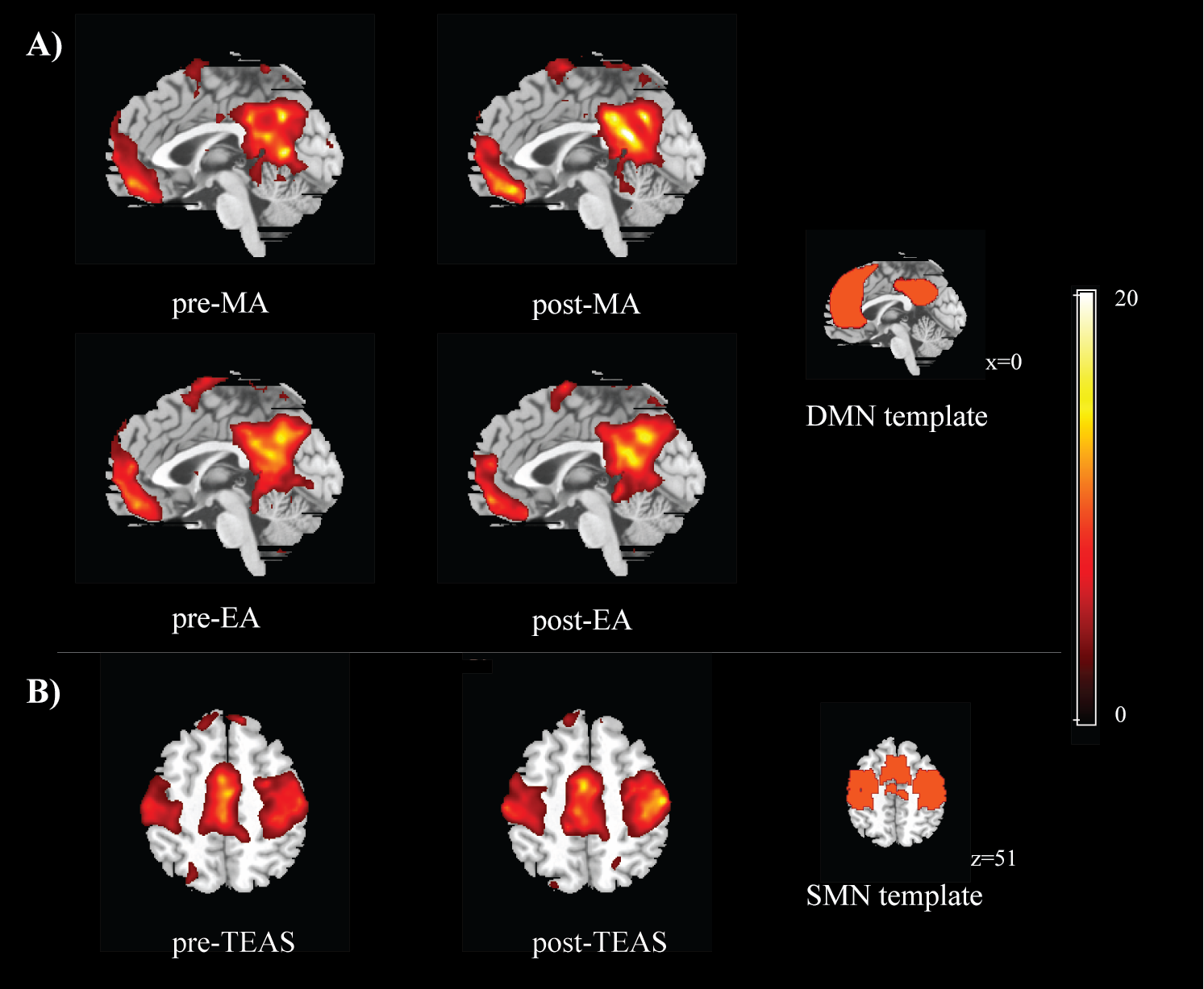


**Fig.S1. Group maps for the DMN and the SMN, before and after acupuncture stimulation.** The best-fit components were selected by using the templates of the DMN and SMN shown in the right line of the graph. The group results of (A) the DMN and (B) the SMN components decomposed by ICA included the pre-MA rest/post-MA rest, the pre-EA rest/post-EA rest and the pre-TEAS rest/post-TEAS rest. The threshold of one sample *t*-test was set as FDR corrected, *P*<0.05, with at least 10 continuous voxels in all group statistics. Color bar indicates T-values.
